# The CYP2E1 inhibitor DDC up-regulates MMP-1 expression in hepatic stellate cells via an ERK1/2- and Akt-dependent mechanism

**DOI:** 10.1042/BSR20130033

**Published:** 2013-06-05

**Authors:** Tianhui Liu, Ping Wang, Min Cong, Youqing Xu, Jidong Jia, Hong You

**Affiliations:** Liver Research Center, Beijing Friendship Hospital, Capital Medical University, Beijing 100050, People's Republic of China

**Keywords:** collagen, cytochrome P450 2E1, diethyldithiocarbamate, matrix metalloproteinase-1, mitogen-activated protein kinases, reactive oxygen species, Akt, protein kinase B, ASH, alcoholic steatohepatitis, CYP2E1, cytochrome P450 2E1, DCF, dichlorofluorescin, DDC, diethyldithiocarbamate, ECM, extracellular matrix, ERK, extracellular signal-regulated kinase, HSC, hepatic stellate cell, MAPK, mitogen-activated protein kinases, MMP-1, matrix metalloproteinase-1, NASH, non-alcoholic steatohepatitis, ROS, reactive oxygen species

## Abstract

DDC (diethyldithiocarbamate) could block collagen synthesis in HSC (hepatic stellate cells) through the inhibition of ROS (reactive oxygen species) derived from hepatocyte CYP2E1 (cytochrome P450 2E1). However, the effect of DDC on MMP-1 (matrix metalloproteinase-1), which is the main collagen degrading matrix metalloproteinase, has not been reported. In co-culture experiments, we found that DDC significantly enhanced MMP-1 expression in human HSC (LX-2) that were cultured with hepatocyte C3A cells either expressing or not expressing CYP2E1. The levels of both proenzyme and active MMP-1 enzyme were up-regulated in LX-2 cells, accompanied by elevated enzyme activity of MMP-1 and decreased collagen I, in both LX-2 cells and the culture medium. H_2_O_2_ treatment abrogated DDC-induced MMP-1 up-regulation and collagen I decrease, while catalase treatment slightly up-regulated MMP-1 expression. These data suggested that the decrease in ROS by DDC was partially responsible for the MMP-1 up-regulation. ERK1/2 (extracellular signal-regulated kinase 1/2), Akt (protein kinase B) and p38 were significantly activated by DDC. The ERK1/2 inhibitor (U0126) and Akt inhibitor (T3830) abrogated the DDC-induced MMP-1 up-regulation. In addition, a p38 inhibitor (SB203580) improved MMP-1 up-regulation through the stimulation of ERK1/2. Our data indicate that DDC significantly up-regulates the expression of MMP-1 in LX-2 cells which results in greater MMP-1 enzyme activity and decreased collagen I. The enhancement of MMP-1 expression by DDC was associated with H_2_O_2_ inhibition and coordinated regulation by the ERK1/2 and Akt pathways. These data provide some new insights into treatment strategies for hepatic fibrosis.

## INTRODUCTION

DDC (diethyldithocarbamate) and its derivatives are widely used in clinical applications. DDC can act as a radioprotective agent and its derivative, disulfiram, is used clinically to treat alcoholism because of its ability to inhibit ALDH (aldehyde dehydrogenase) [1,2]. It has been reported that low doses of DDC protect against liver injury induced by many hepatotoxic agents *via* inhibitory activity on drug metabolizing enzymes and antioxidant effects in rats [3]. DDC or disulfiram (pro-drug of DDC) is widely used for inhibition of CYP2E1 (cytochrome P450 2E1) in human studies because of its selectivity and relative lack of toxicity [4].

CYP2E1 is highly expressed in the liver where it has a great capacity to produce ROS (reactive oxygen species) [5,6], which may lead to oxidative stress. Hepatic CYP2E1 has been linked to the pathogenesis of ASH (alcoholic steatohepatitis) [7], hepatic steatosis and NASH (non-alcoholic steatohepatitis) [8–10]. As the common complication of most chronic liver diseases, including ASH and NASH, liver fibrosis is triggered by chronic liver injury and develops from a series of events. After liver injury, damaged hepatocytes release ROS which, subsequently, have some effect on HSC (hepatic stellate cells). Basic research has demonstrated that HSCs are the key fibrogenic cells through synthesizing and secreting ECM (extracellular matrix) [11]. Repeated hepatocyte injury and uncontrolled repair processes result in liver fibrosis characterized by substantial deposition of ECM [12–15]. *In vitro* studies demonstrated that elevated H_2_O2, generated by hepatocytes expressing CYP2E1 increased the intracellular concentration of H_2_O_2_ in HSCs, which subsequently activated HSCs and induced collagen I production [16,17].

Since CYP2E1-derived ROS are involved in fibrogenesis, the inhibitory effects of antioxidants and CYP2E1 inhibitors on liver fibrosis have been investigated both *in vivo* and *in vitro* [18–21]. It has been demonstrated that DDC directly suppresses collagen I protein by decreasing ROS [16]. However, it is not clear whether DDC modulates other proteins or signalling pathways which may contribute to the decrease in collagen I protein. Liver fibrosis is characterized by excessive deposition of type I collagen fibrils in the Disse's space [22]. Changes in collagen I protein may be due to transcription of collagen mRNA in combination with protein degradation. During the degradation process, MMP-1 (matrix metalloproteinase-1) plays the most important role in fibrolysis, especially by degrading excessive deposition of type I collagen [23]. Therefore we hypothesized that DDC may decrease collagen I through modulation of MMP-1 expression.

In this study, a co-culture model was established, based on the co-incubation of the human hepatic cell lines (C3A), expressing CYP2E1 (C3A-2E1 cells) or non-CYP2E1 expressing cells (C3A cells), with human HSC (LX-2). We examined the effect of DDC on MMP-1 expression and enzyme activity in LX-2 cells. In addition, we investigated the role of H_2_O_2_, and other molecular mechanisms that may regulate MMP-1 expression.

## MATERIAL AND METHODS

### Materials

DDC, T3830 [Akt (protein kinase B) inhibitor], catalase, and H_2_O_2_ were purchased from Sigma-Aldrich (St. Louis). U0126 [ERK1/2 (extracellular signal-regulated kinase) inhibitor] was purchased from Promega (Madison). SB203580 (p38 inhibitor) was purchased from Invitrogen. Vitamin E was purchased from SUPELCO. Anti-CYP2E1 (cat. no. ab53945) was purchased from Abcam. Anti-p38 (cat. no. 4511) and anti-phospho-p38 (cat. no. 9212) were purchased from CST. Anti-MMP-1 (cat. no. MAB901), anti-ERK1/2 (cat. no. AF1576), anti-phospho-ERK1/2 (cat. no. MAB1018), anti-Akt (cat. no. AF887) and anti-phospho-Akt (cat. no. MAB2055) were purchased from R&D. Anti-MMP-1 (active form only, cat. no. MAB3223) was purchased from Millipore. Anti-col1A2 (cat. no. 14695-1-AP) was purchased from Proteintech.

### Cell transfection and selection

C3A cells were transfected with the pCI-neo (without the *CYP2E1* gene) or pCI-2E1 plasmid (with the *CYP2E1* gene) using Fugene HD (Roche Diagnostic). Cells were then selected with 0.8 mg/ml geneticin (G-418); colonies formed from the surviving cells were grown to large scale, and subjected to real-time (RT)-PCR and Western blotting analysis to determine CYP2E1 expression.

### Cell culture

**Figure 1:**
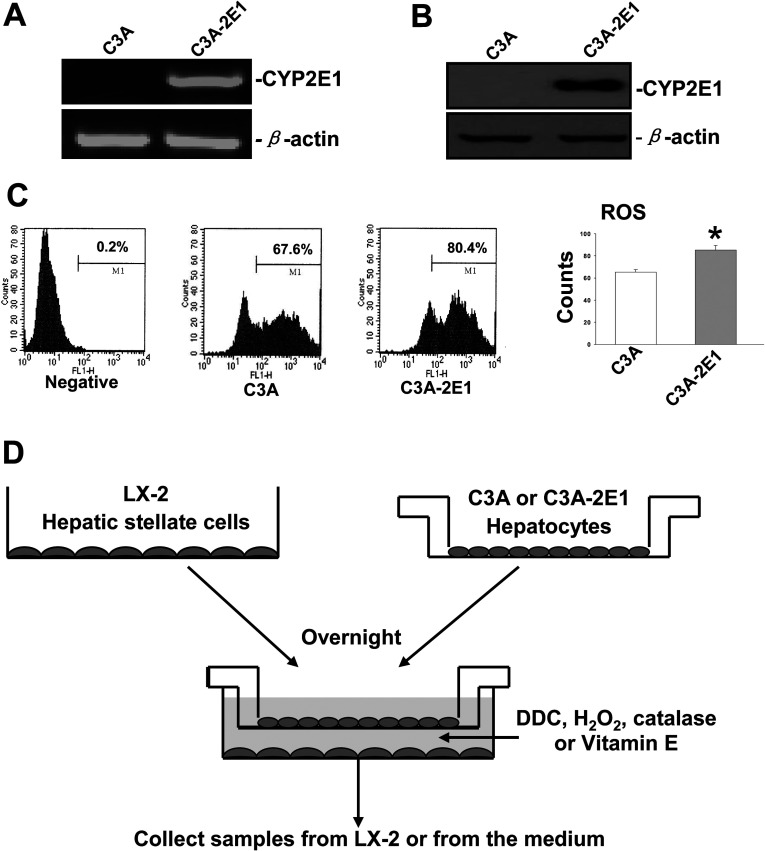
Establishment and identification of C3A-2E1 cell lines expressing human CYP2E1 and the scheme of the co-culture model (**A**) RT-PCR analysis of total RNA to detect CYP2E1 expression in C3A cells transfected with pCI-neo (lane 1) and pCI–2E1 plasmids (lane 2). (**B**) 50 μg aliquots of total protein extracts from C3A cells were subjected to Western blotting analysis with polyclonal rabbit anti-human CYP2E1 antibody and β-actin antibody as a loading control. (**C**) Intracellular ROS levels in C3A cells transfected with pCI-neo and pCI–2E1 plasmids. (**D**) Scheme of the co-culture model of C3A cells, expressing (C3A-2E1) or not expressing (C3A) CYP2E1 with LX-2 cells. Cells were plated at a 5:1 ratio of C3A cells/LX-2. LX-2 cells were cultured alone overnight, then the cell culture inserts containing C3A cells were transferred together with the culture medium to the plates containing the LX-2 cells; this defined the beginning of the co-culture period. Samples of LX-2 cells or culture medium were collected at different time points.

The model used in most experiments is based on co-culturing C3A cell lines, expressing CYP2E1 (C3A cells) or not (C3A-2E1 cells), with LX-2 cells (Figure 1C). Cells were co-cultured using cell culture inserts (3 μm pore size) to separate the cell populations; the LX-2 cells were plated on the bottom, and the C3A cells were plated on the insert to create a gravity gradient of the released mediators. A 5:1 ratio of C3A/LX-2 was chosen, because this was considered to represent the ratio of parenchymal/non-parenchymal cells in the liver. After overnight incubation of LX-2 cells in DMEM (Dulbecco's modified Eagle's medium) supplemented with 10% (v/v) fetal bovine serum, the LX-2 medium was discarded, the cell culture inserts containing the overnight-incubated C3A or C3A-2E1 cells were transferred, and the medium from these cells was added to the co-culture systems. At this time, DDC, H_2_O_2_, catalase, vitamin E or inhibitors for signalling pathways were added (*t*=0 h). After 24 h co-culture, the cells and supernatants were collected for different assays. For detecting the secreted protein levels in the culture medium, the serum-free medium was used for the co-culture systems.

### Measurement of intracellular ROS

ROS levels were determined by measuring the oxidative conversion of DCFH-DA (2′,7′-dichlorofluorescin diacetate) to the fluorescent compound DCF (dichlorofluorescin). Fluorescence was measured by flow cytometry on an FACScan flow cytometer, using an excitation wavelength of 488 nm and an emission wavelength of 525 nm. Finally, ROS generation was quantified by the percentage of DCF-positive cells of 10000 cells. All measurements were performed in triplicate.

### Measurement of collagen I in cell culture medium by ELISA

The ELISA kit for human collagen I was purchased from BlueGene. The levels of collagen I secreted from LX-2 cells were quantified according to the manufacturer's guidelines. All samples were assayed in duplicates. The measurements were repeated at least three times, with independent cell batches.

### RT-PCR for CYP2E1 and MMP-1

Total RNA was isolated from C3A or LX-2 cells using Trizol® reagent (Invitrogen) according to the manufacturer's instructions. The yield of total RNA was quantified and equivalent amounts of total RNA (2 μg) were reverse-transcribed into single-stranded cDNA. PCR was performed on 100 ng reverse-transcribed total RNA from each sample. The primers were: (1) *CYP2E1*, forward: 5′-TCATAGCCGACATCCTCTTC-3′, reverse: 5′-GTTGTGCTGGTGGTCTCTGT-3′; (2) *β-actin*, forward: 5′-GGGCATGGGTCAGAAGGATT-3′ reverse: 5′-GAGGCGTACAGGGATAGCAC-3′ and (3) *MMP-1*, forward: 5′-AGCTAGCTCAGGATGACATTGATG-3′ reverse: 5′-TCAGAAAGAGCAGCATCGATATG-3′.

### Quantification of MMP-1 expression by RT-PCR

To monitor the modulation of *MMP-1* mRNA levels, a quantitative RT-PCR approach was used. Equal amounts of cDNA were subjected to PCR in the presence of SYBR green dye, with the ABI Power SYBR Green PCR Master Mix kit (Applied Biosystems) on an ABI Prism 7300 Sequence Detector (Applied Biosystems). PCR without template was used as a negative control. *β-actin* mRNA was used as an internal control. The primers used were: *MMP-1* forward: 5′-GATGAAGTCCGGTTTTTCAAAG-3′, reverse: 5′-GGGGTATCCGTGTAGCACCAT-3′; and *β-actin* forward: 5′-AGCAAGCAGGAGTATGACG-3′, reverse: 5′-AAAGGGTGTAACGCAACTAA-3′. PCR was performed with 50 cycles of 15 s at 95°C and 60 s at 60°C after a 2 min initial denaturation at 95°C. Each sample was normalized by the difference in CT (critical thresholds) between the target gene and *β-actin*, and relative to the control, the amount of target was given as 2^−ΔΔCT^. All experiments were performed independently three times, and the average was used for comparison.

### Casein zymography for MMP-1

For assessment of MMP-1 activity, casein zymography was performed [24,25]. Thirty μg of LX-2 lysates or 20 μl aliquots of 10-fold concentrated culture medium passed through centricon columns (molecular mass cut-off of 50 kDa, Millipore) with 5×loading buffer [0.25 M Tris pH 6.8, 50% (v/v) glycerol, 5% (w/v) SDS, bromophenol blue] and were run on Novex 4–16% Zymogram (Blue Casein) Gel (Invitrogen) at 125 V for 2 h (buffer 25 mM Tris, 190 mM glycine, 0.1% SDS). Gels were incubated in 2.5% (v/v) Triton X for 1 h at room temperature with agitation and then collagenase buffer (55 mM Tris base, 200 mM sodium chloride, 5 mM calcium chloride, 0.02% Brij, pH 7.6) for 30 min. Gels were then incubated for 24 h in fresh collagenase buffer at 37°C. Densitometric analysis of zymography gels was performed with the Quantity One® software package.

### Phospho-proteome profiling

Cells were rinsed with PBS and immediately solubilized in lysis buffer at 4°C for 30 min. Following microcentrifugation at 14000 ***g*** for 5 min, supernatants were transferred into a new tube and protein concentrations were determined using the Pierce Protein assay kit (Pierce). 200 μg of LX-2 lysates were diluted and incubated with the Human Phospho-MAPK (mitogen-activated protein kinases) Array Kit (Proteome Profiler™; R&D) according to the manufacturer's instructions. Phospho-MAPK Array data were developed on X-ray films following exposure to chemiluminescent reagents (Pierce).

### Western blot analyses

To monitor CYP2E1, MMP-1, collagen I and MAPKs protein levels, Western blotting was used. Protein mixtures were separated on SDS/12%PAGE electrophoresis gels and transferred onto nitrocellulose membranes (Amersham Biosciences). Membranes were blocked and then incubated with anti-CYP2E1 (1:1000), anti-MMP-1 (1:500), anti-ERK1/2 (1:1000), anti-phospho-ERK1/2 (1:500), anti-p38 (1:1000), anti-phospho-p38 (1:1000), anti-Akt (1:2000), anti-phospho-Akt (1:1000), anti-COL1A2(1:800) and anti-β-actin (1:5000) at 4°C overnight. After extensive washing, membranes were incubated with the secondary antibody for 60 min, followed by extensive washes. Specific antibody–antigen complexes were detected with the ECL Western blotting detection kit (Pierce). All experiments were performed independently at least three times, and the protein expression was quantified by densitometric analysis of immunoblots using the Quantity One software.

### Statistical analysis

All values are indicated as the mean±S.E.M. Two-group comparisons were carried out by the Student's *t* test. Comparisons of means of three or more groups were carried out by ANOVA, and then by LSD (least significant difference) test when the variance was equal and by Dunnett T3 test when the variance was unequal. A difference with *P*<0.05 was considered statistically significant.

## RESULTS

### Establishment of C3A-2E1 cell lines expressing human CYP2E1 and the co-culture model

C3A cells, derived from HepG2 cells, are a human hepatoblastoma cell line that maintains several liver functions but do not express CYP2E1. Since CYP2E1 is expressed in normal hepatocytes, we established a C3A cell model expressing CYP2E1. C3A cells were transfected with pCI-neo plasmid (without CYP2E1) or with pCI-2E1 plasmid (with CYP2E1). RT-PCR results demonstrated that C3A cells transfected with pCI-neo (C3A) did not express CYP2E1 (Figure 1A, lane 1), in contrast with C3A cells transfected with pCI–2E1 (C3A-2E1), which expressed significant amounts of CYP2E1 (Figure 1A, lane 2). Western blotting analysis showed a clear ~54 kDa band, representing CYP2E1 expression (Figure 1B, lane 2), whereas the C3A cells did not express detectable levels of CYP2E1 (Figure 1B, lanes 1). ROS levels were measured in both cell lines, and were found to be elevated by 20% in C3A-2E1 cells compared with C3A cells (Figure 1C). These data indicated that C3A-2E1 cells expressed CYP2E1 and produced more ROS. C3A or C3A-2E1 cells were then co-cultured with LX-2 cells (see Figure 1D for the co-culture model scheme).

### CYP2E1 inhibitor DDC lowered intracellular ROS in LX-2 cells and inhibited the production and secretion of collagen I from LX-2 cells

**Figure 2:**
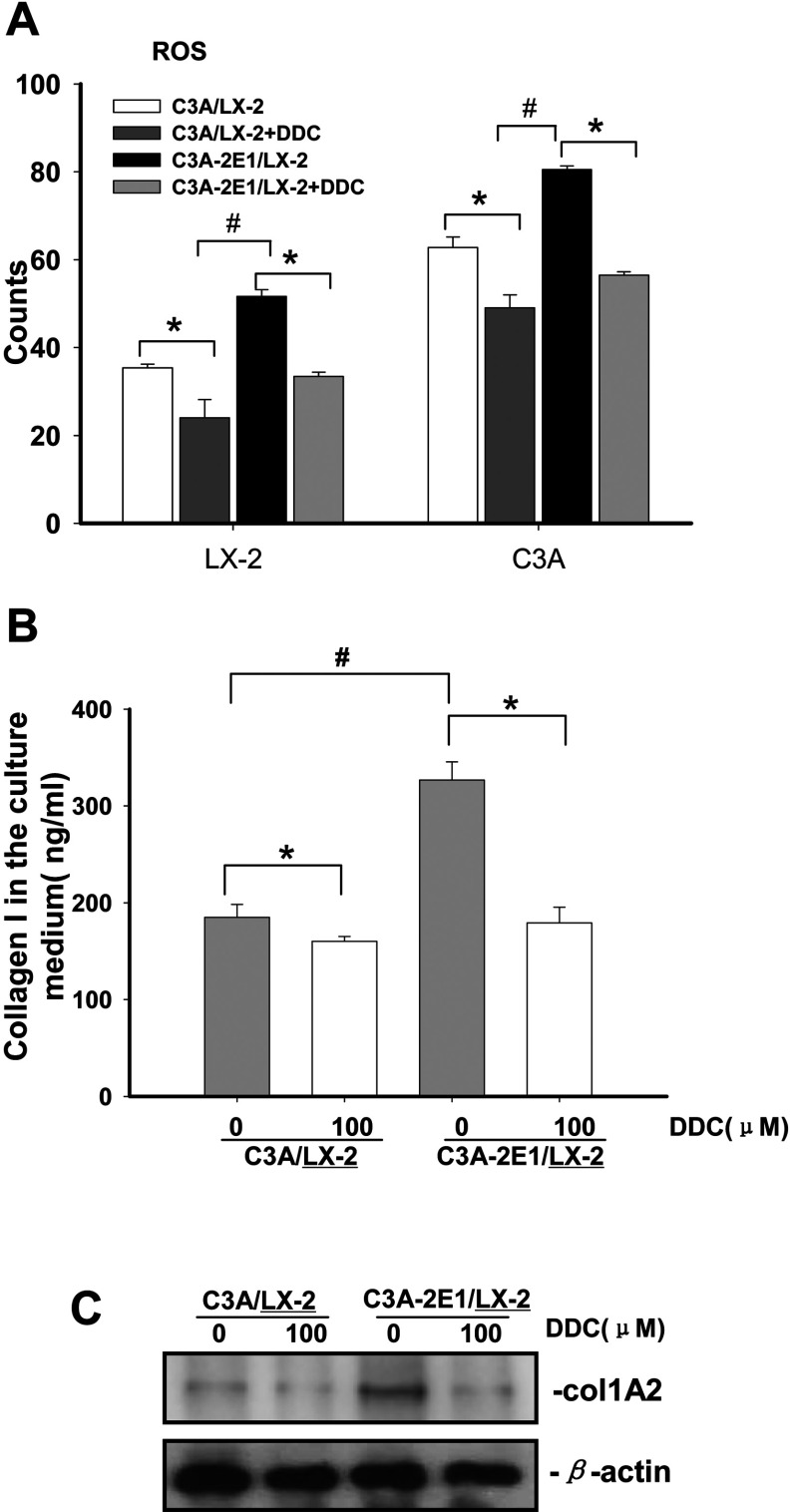
DDC lowered ROS and collagen I protein in LX-2 cells The co-culture systems were treated with or without 100μM DDC for 24 h. (**A**) Both C3A cells and LX-2 cells were harvested and incubated with 10 μM DCFH-DA at 37°C for 15 min in the dark. The fluorescence intensity was monitored by flow cytometry using an excitation wavelength of 488 nm and an emission wavelength of 525 nm. Representative results are shown.* *P*<0.05 compared with untreated C3A/LX-2 or C3A-2E1/LX-2. # *P*<0.05 compared with C3A/LX-2. (**B**) Levels of collagen I in the culture medium were assayed by ELISA.*P<0.05 compared with untreated C3A/LX-2 or C3A-2E1/LX-2. # *P*<0.05 compared with C3A/LX-2. (**C**) Levels of collagen I in LX-2 cells were assayed by Western blotting.

To investigate whether CYP2E1-derived products such as ROS from C3A-2E1 cells can diffuse and create a pro-oxidant stress in LX-2 cells and the effect of DDC on ROS, we measured the levels of ROS both in C3A or C3A-2E1 cells and in LX-2 cells after 24 h of co-culture (with or without 100 μM DDC). As shown in Figure 2A, ROS were elevated in C3A-2E1 cells when compared with C3A cells (*P*<0.05), and were reduced in both C3A and C3A-2E1 cells following DDC treatment. Meanwhile, ROS was elevated in LX-2 cells co-cultured with C3A-2E1 cells when compared with those co-cultured with C3A cells (*P*<0.05). ROS were reduced in LX-2 cells co-cultured with both C3A and C3A-2E1 cells following DDC treatment (*P*<0.05).

Since ROS mediate paracrine stimulation of collagen I protein synthesis, and DDC attenuated ROS production in LX-2 cells, the inhibitory effect of DDC on collagen I was assessed. The co-cultures were treated with 100 μM DDC for 24 h. The incubation media were collected and analysed for collagen I by ELISA. The collagen I protein increased in LX-2/C3A-2E1 co-culture compared with the C3A co-culture, and decreased in both systems treated with DDC (Figure 2B). The LX-2 cells were analysed by Western blotting for intracellular collagen I. The collagen type I protein in LX-2 cells increased in the C3A-2E1 co-culture compared to the C3A co-culture, and was reduced in both systems following DDC treatment (Figure 2C).

### DDC up-regulates MMP-1 expression in LX-2 cells

**Figure 3:**
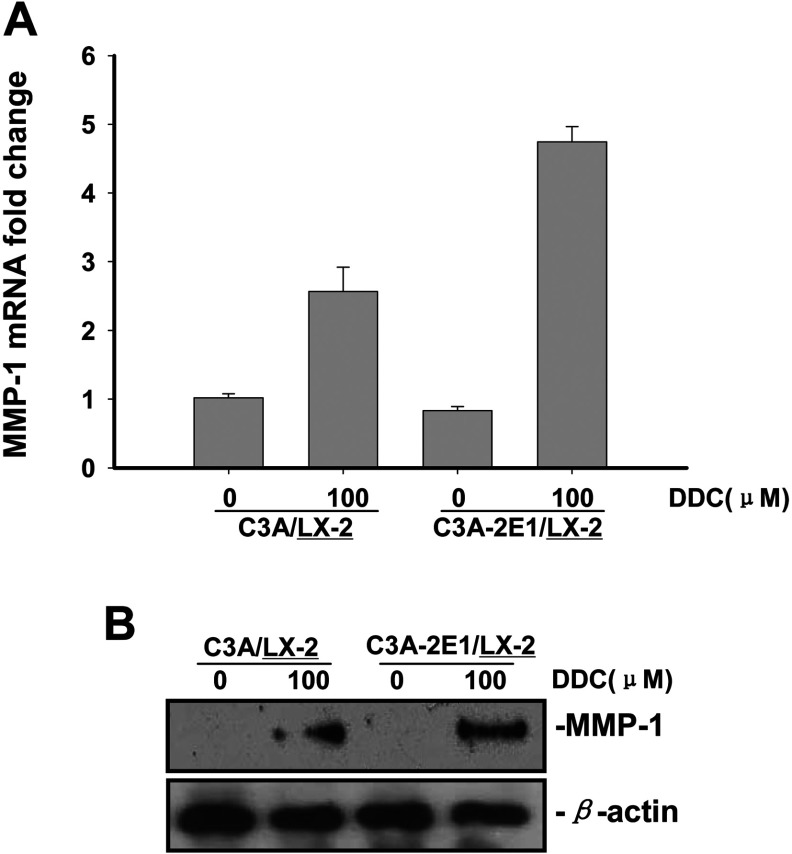
DDC up-regulates MMP-1 expression in LX-2 cells Co-culture systems were treated with 100 μM DDC for 24 h. LX-2 cells and the culture medium were collected and analysed. (**A**) Quantitative RT-PCR results of *MMP-1* expression in LX-2 cells. The results were normalized with β-actin and expressed as fold increase relative to the control in each experiment. (**B**) 20 μg aliquots of total protein extracts from LX-2 cells were subjected to western blotting analysis with anti-MMP-1 antibody (MAB 901) and β-actin antibody as a loading control, as described in the Materials and Methods section.

The co-cultures were treated with 100 μM DDC for 24 h. As shown in Figure 3A, *MMP-1* mRNA expression in LX-2 cells co-cultured with C3A was up-regulated 2.5-fold by DDC, compared with untreated LX-2 cells co-cultured with C3A cells. *MMP-1* mRNA expression in LX-2 cells co-cultured with C3A-2E1 was up-regulated 4.6-fold by DDC, compared with untreated LX-2 cells co-cultured with C3A-2E1 cells. In addition, DDC caused an up-regulation of the MMP-1 proenzyme in LX-2 cells under both co-culture systems. Note that the up-regulation of MMP-1 was higher in the C3A-2E1 co-culture system than in the C3A co-culture system. These data suggested that the inhibition of CYP2E1 activity by DDC only plays a small role in MMP-1 up-regulation (Figure 3B).

### DDC up-regulates MMP-1 expression in a dose-dependent manner and results in greater enzyme activity

**Figure 4:**
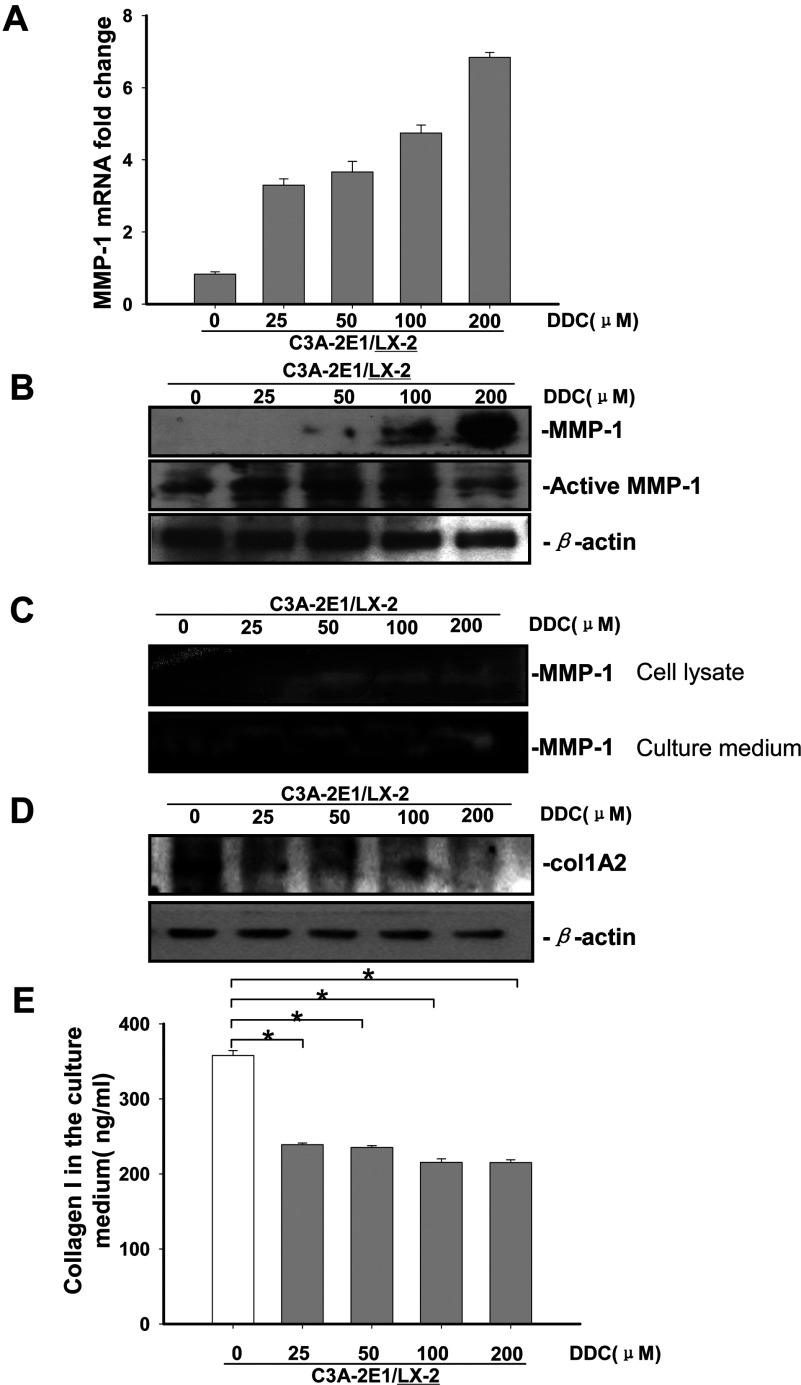
DDC up-regulates MMP-1 expression in a dose-dependent manner and lowered collagen I in LX-2 cells Co-culture systems were treated with 25, 50, 100 and 200 μM DDC for 24 h. LX-2 cells and the culture medium were collected and analysed. (**A**) Quantitative RT-PCR results of *MMP-1* expression in LX-2 cells. The results were normalized with β-actin and expressed as fold increase relative to the control in each experiment. (**B**) 20 μg aliquots of total protein extracts from LX-2 cells were subjected to Western blotting analysis with anti-MMP-1 antibody (MAB 901 and MAB 3223 for active form only) and β-actin antibody as a loading control, as described in the Materials and Methods section. (**C**) 30 μg of LX-2 lysates or 20 μl aliquots of 10-fold concentrated culture medium with 5×loading buffer were assayed by Casein Zymography for MMP-1 activity. (**D**) Levels of collagen I in LX-2 cells were assayed by western blotting. (**E**) Levels of collagen I in the culture medium were assayed by ELISA. *P<0.05 compared with untreated C3A-2E1/LX-2.

The co-cultures were treated with 25, 50, 100 and 200 μM DDC for 24 h. As shown in Figure 4(A), *MMP-1* mRNA expression in LX-2 cells was up-regulated in the presence of 25 μM (3.2-fold), 50 μM (3.5-fold), 100 μM (4.6-fold) and 200 μM (6.8-fold) DDC, compared with untreated LX-2 cells co-cultured with C3A-2E1 cells. These data suggest that the positive effect of DDC on *MMP-1* expression in LX-2 cells was dose-dependent in the tested range. In addition, the proenzyme of MMP-1 was up-regulated in a dose-dependent manner in the presence of 50, 100 and 200 μM DDC, compared with untreated LX-2 cells co-cultured with C3A-2E1 (Figure 4B).

We next analysed the activity of MMP-1 both in the LX-2 cells and the culture medium to investigate whether increased gene expression results in greater enzymatic activity. The Western blotting results showed that the active enzyme of MMP-1 was up-regulated in a dose-dependent manner in the presence of 50, 100 and 200 μM DDC, compared with untreated LX-2 cells co-cultured with C3A-2E1 (Figure 4B). Analysis of MMP-1 concentration by casein zymography and Western blotting has been shown to correlate closely [26,27]. As shown in Figure 4(C), casein zymography demonstrated that MMP-1 activity both in LX-2 cells and in the culture medium closely paralleled levels of gene expression. Levels of collagen I in LX-2 cells and culture medium were analysed by Western blotting and ELISA. As shown in Figures 4(D) and 4(E), the production and secretion of collagen I protein was significantly inhibited by DDC.

### Inhibition of ROS by DDC partially contributes to DDC-induced MMP-1 up-regulation

Since MMP-1 protein in LX-2 cells was up-regulated by DDC in both co-culture systems (with and without CYP2E1), these data suggested that MMP-1 up-regulation by DDC did not merely depend on the inhibition of CYP2E1 activity. However, DDC reduced ROS production in both C3A and C3A-2E1 cells, which ameliorated the pro-oxidant stress in LX-2 cells. We investigated next whether the inhibition of ROS by DDC was involved in the DDC-dependent up-regulation of MMP-1.

**Figure 5:**
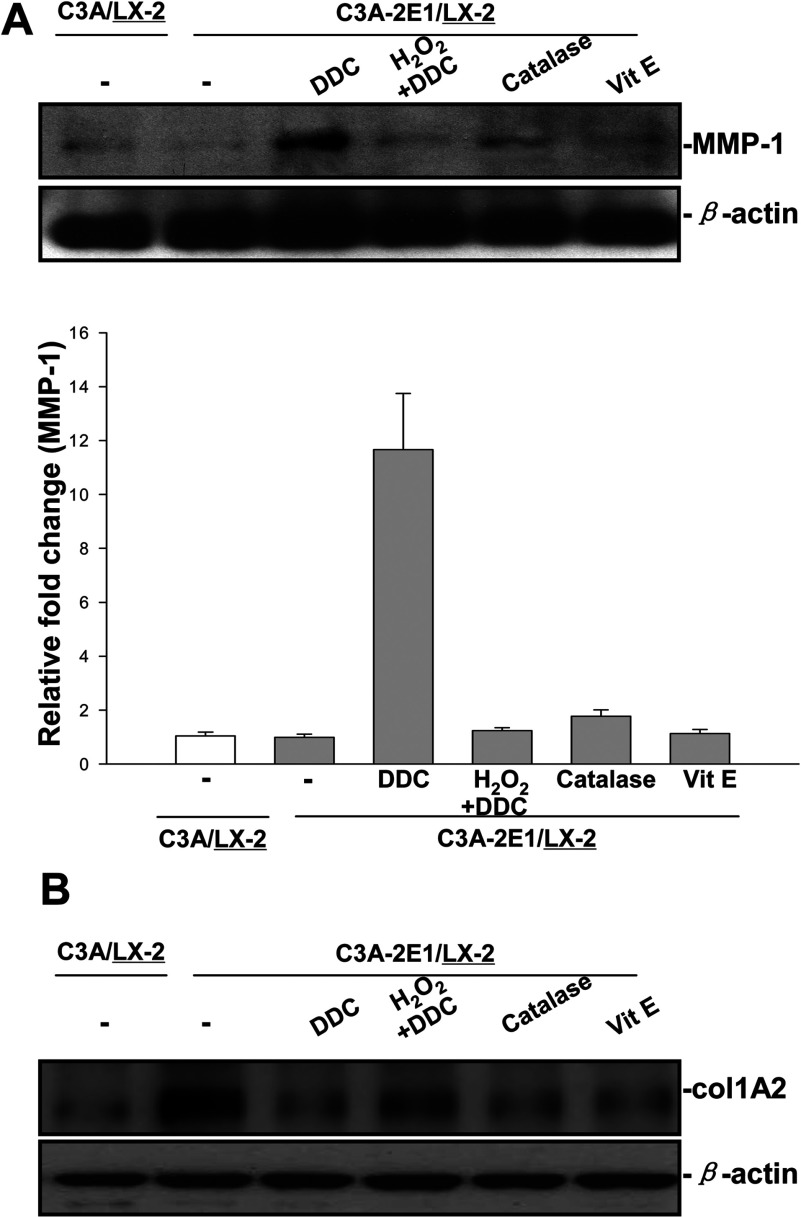
DDC up-regulates MMP-1 expression in an H_2_O_2_-associated manner Co-cultures were treated with 100 μM DDC, 2000 Units catalase, 25 μM vitamin E and 5μM H_2_O_2_ combined with 100 μM DDC for 24 h. Cell lysates from the LX-2 cells were collected and analysed. (**A**) 20 μg aliquots of total protein extracts from LX-2 cells were subjected to Western blotting analysis with anti-MMP-1 antibody (MAB 901) and β-actin antibody as a loading control, as described in the Materials and Methods section. (**B**) Levels of collagen I in LX-2 cells were assayed by Western blotting.

Compared with untreated LX-2 cells, MMP-1 protein in LX-2 cells increased after DDC treatment. H_2_O_2_ treatment reduced this DDC-induced up-regulation. In addition, catalase alone also slightly up-regulated MMP-1. However, no significant increase of MMP-1 was observed in LX-2 cells after vitamin E treatment (Figure 5A). These data suggested that the decrease in ROS by DDC was partially responsible for MMP-1 up-regulation. Compared with untreated LX-2 cells co-cultured with C3A-2E1 cells, collagen-1 protein in LX-2 cells decreased after DDC, catalase and vitamin E treatment. Compared with DDC treated LX-2 cells, H_2_O_2_ treatment attenuated the DDC-mediated decrease in collagen I (Figure 5B).

### Phospho-proteomic profiling of LX-2 cells co-cultured with C3A-2E1 cells after DDC treatment

**Figure 6:**
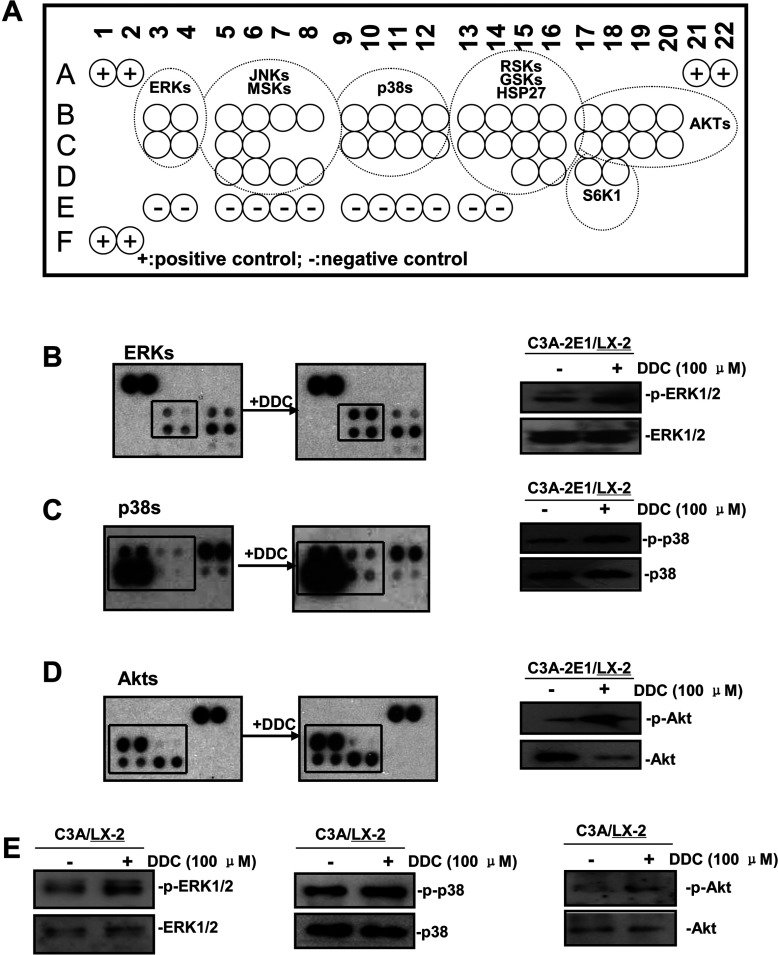
Phospho-proteome profiling results of LX-2 cells co-cultured with C3A–CYP2E1 cells with or without DDC treatment and detection of the effect of DDC on intracellular kinases in LX-2 cells of co-cultures 200 μg of total cell lysates from LX-2 cells co-cultured with C3A-2E1 cells with or without 100 μM DDC for 1 h were incubated with membranes of the human phospho-MAPK Array Kit according to the manufacturer's instructions. Phospho MAPK Array data were developed on X-ray films following exposure to chemiluminescent reagents. 20 μg aliquots of total cell lysates from LX-2 cells were subjected to Western blotting analysis. (**A**) Template showing the location of MAPK antibodies spotted onto the human phospho-MAPK Array Kit. (**B**) The activation status of ERK1/2 in LX-2 cells co-cultured with C3A-2E1 cells after DDC treatment. (**C**) The activation status of p38 in LX-2 cells co-cultured with C3A-2E1 cells after DDC treatment. (**D**) The activation status of Akt in LX-2 cells co-cultured with C3A-2E1 cells after DDC treatment. (**E**) The activation status of ERK1/2, p38 and Akt in LX-2 cells co-cultured with C3A cells after DDC treatment.

Since the decrease in ROS by DDC was only partially responsible for MMP-1 up-regulation, we next analysed possible signal transduction pathways involved in DDC-dependent up-regulation of MMP-1. The human Phospho-MAPK Array Kit, is a rapid, sensitive and semiquantitative tool that is able to identify the levels of phosphorylation of multiple intracellular kinases (Figure 6A). LX-2 cells were co-cultured with C3A-2E1 cells, with or without 100 μM DDC, for 1 h.

ERK1/2, p38 and Akt were highly activated after 1 h DDC treatment (Figures 6B–6D). To further confirm the phospho-proteome profiling results, the activation of ERK1/2, p38 and Akt were analysed by Western blotting (Figures 6B–6D). To determine if similar mechanisms were involved in LX-2 cells co-cultured with C3A cells (without CYP2E1), we investigated the effect of DDC on the activation of ERK1/2, p38 and Akt in LX-2 cells co-cultured with C3A cells. The results showed that DDC significantly increased the activation of ERK1/2, p38 and Akt in LX-2 cells of both co-cultures (Figure 6E), suggesting that multiple MAPK signalling pathways may contribute to DDC-induced MMP-1 up-regulation.

### DDC up-regulates MMP-1 through activating ERK1/2 and Akt

**Figure 7:**
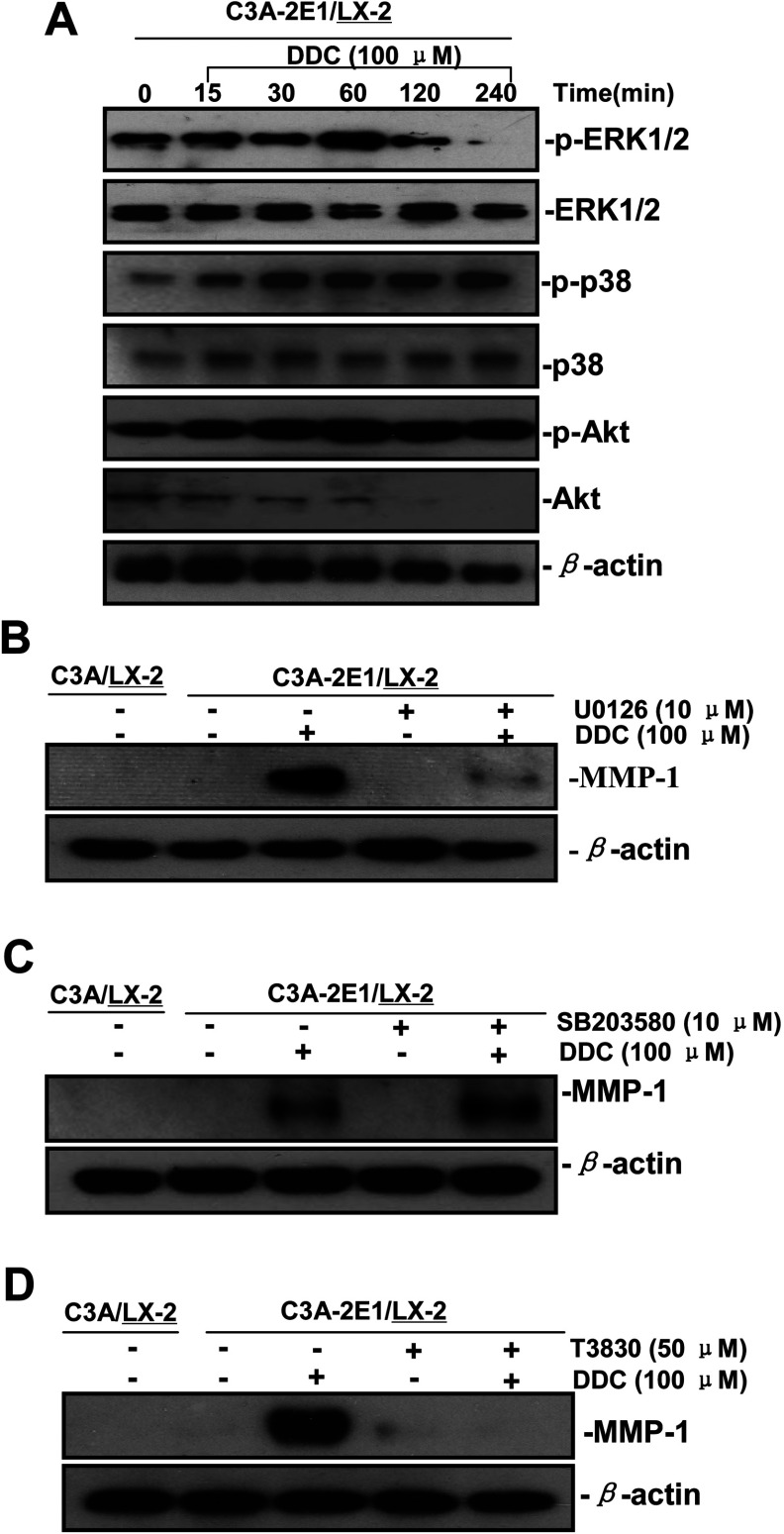
DDC up-regulates MMP-1 through ERK1/2 and Akt activation (**A**) Co-cultures were treated with 100 μM DDC for the indicated time. Phosphorylation of ERK1/2, p38 and Akt were determined by Western blotting analysis. The corresponding non-phosphorylated ERK1/2, p38, Akt and β-actin were used for protein loading control. (**B**) Co-cultures were treated with or without 100 μM DDC for 24 h in the presence or absence of ERK1/2 inhibitor (U0126, 10 μM). MMP-1 protein levels were analysed by Western blotting. (**C**) Co-cultures were treated with or without 100 μM DDC for 24 h in the presence or absence of p38 inhibitor (SB203580, 10 μM). MMP-1 protein levels were analysed by Western blotting. (**D**) Co-cultures were treated with or without 100 μM DDC for 24 h in the presence or absence of Akt inhibitor (T3830, 50 μM). MMP-1 protein levels were analysed by Western blotting.

After demonstrating that Akt, ERK1/2 and p38 may contribute to DDC-induced MMP-1 up-regulation, we examined the effect of DDC on ERK1/2, p38 and Akt activity. To this end, the co-cultures were treated with or without DDC. As shown in Figure 7(A), the phosphorylation of ERK1/2 was induced 15 min after treatment, peaked at 60 min, and sharply decreased to below control values by 240 min. DDC induced the phosphorylation of p38 MAPK and Akt at 15 min, which peaked at 60 min and was sustained for up to 240 min.

To investigate the significance of these effects on the DDC-induced MMP-1 expression, co-cultures were treated with or without DDC for 24 h in the presence or absence of ERK1/2 inhibitor), p38 inhibitor (SB203580) or Akt inhibitor (T3830). The inhibition of ERK1/2 and Akt effectively blocked DDC-induced MMP-1 up-regulation and collagen I decrease. In contrast, after treating cells with SB203580, DDC-induced expression of MMP-1 was higher than the control values. These results indicate that ERK1/2 and Akt activation contributed to MMP-1 up-regulation by DDC (Figures 7B–7D).

### p38 inhibitor SB203580 improves the up-regulation of MMP-1 by DDC through stimulating ERK1/2

**Figure 8:**
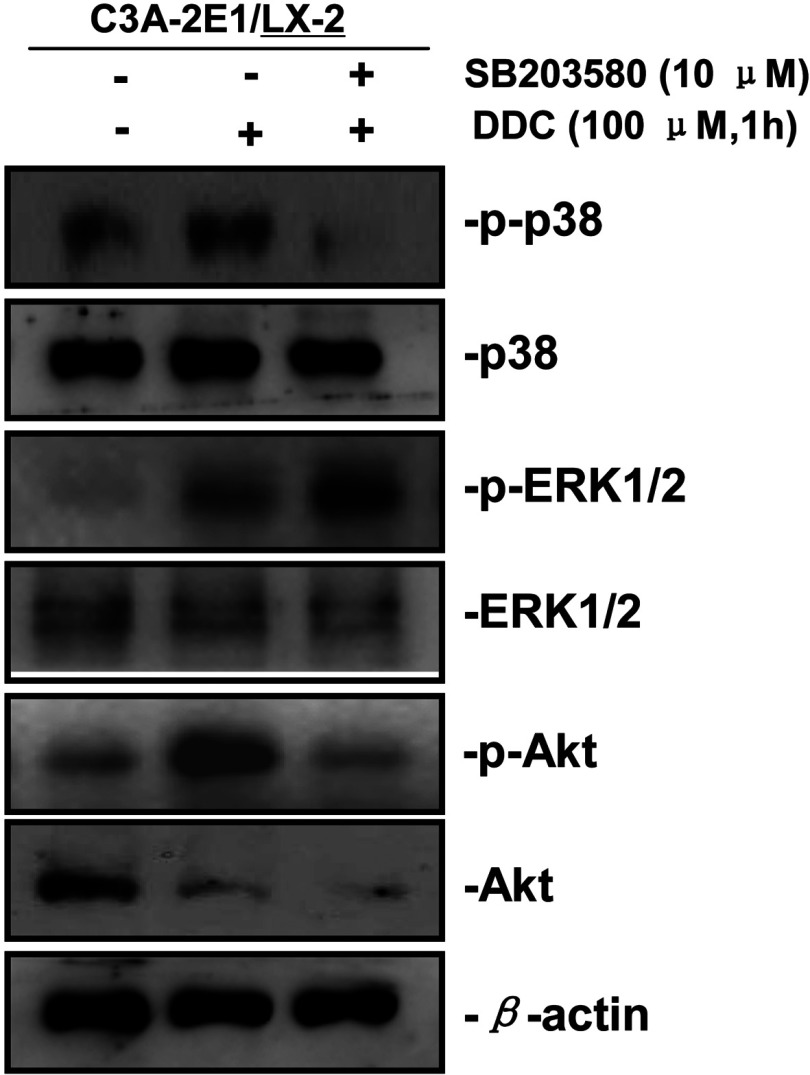
p38 inhibitor SB203580 improves the up-regulation of MMP-1 by DDC through stimulating ERK1/2 Co-cultures were treated with or without 100 μM DDC for 1 h in the presence or absence of p38 inhibitor (SB203580, 10 μM). Phosphorylation of ERK1/2, p38 and Akt in LX-2 cells of co-cultures were determined by Western blotting analysis.

Since the co-treatment of p38 inhibitor, SB203580, with DDC resulted in a more pronounced increase in MMP-1 protein level, we investigated the mechanisms that may contribute to this effect. Co-cultures were treated with or without DDC in the presence or absence of the p38 inhibitor (SB203580). As shown in Figure 8, SB203580 significantly activated ERK1/2, whereas it inhibited Akt activity. These data indicate that p38 inhibitor SB203580 improved MMP-1 up-regulation by DDC through stimulating ERK1/2.

## DISCUSSION

Liver fibrosis is a common response to chronic liver injury, regardless of its nature [viral infection, alcohol abuse, polymetabolic syndrome, drug toxicity, autoimmune disorders or metal (especially iron or copper) overload] and is characterized by excessive deposition of ECM components [28]. The development of liver fibrosis is a multicellular process involving paracrine signalling between resident liver cells and non-resident inflammatory cells. The central event in the initiation of liver fibrogenesis relates to the activation of HSC in the area of tissue necrosis and inflammation for which ROS play an important role. Direct stimulation of HSC proliferation and collagen synthesis by products generated from hepatocytes has been shown to contribute to iron- and alcohol-related fibrosis. Production of pro-oxidant agents or lipid peroxidation products in parenchymal cells can lead to an increase in collagen production by HSC [29,30]. Results with the co-culture (HepG2–HSC) model *in vitro* indicated that CYP2E1-derived ROS from hepatocytes could increase collagen protein synthesis by HSC [16,17]. *In vivo*, fibrosis has been associated with free radical production in hepatocytes located near HSC, either from iron overload-induced [30,31] or CCl4-induced liver injury [32]. Prevention of fibrosis by antioxidants and the CYP2E1 inhibitor (DDC) indicated that ROS play an important role in the CYP2E1-dependent up-regulation [17]. In the present study, we investigated the possible anti-fibrotic mechanisms of DDC. Our data showed that DDC significantly up-regulates the expression of MMP-1 in LX-2 cells, which results in greater enzyme activity of MMP-1. The enhancement of MMP-1 expression by DDC was associated with H_2_O_2_ inhibition and coordinated regulation by the ERK1/2 and Akt pathways.

To investigate the possible anti-fibrotic mechanism of DDC, we developed a co-culture model, which was based on co-incubation of human hepatic C3A-2E1 cells, which overexpress CYP2E1, with human hepatic LX-2 cells. Hepatocytes will lose CYP2E1 expression shortly after their separation from animal liver and culturing. C3A, derived from HepG2, do not express CYP2E1, and can therefore be used as CYP2E1 knock-down cells. In agreement with Nieto *et al*. [16], our results showed that C3A-2E1 cells could produce more ROS than C3A cells, which subsequently increased the intracellular concentration of ROS and collagen I in LX-2 cells. However, in this cell model, C3A cells (without CYP2E1) have a high basal level of ROS. It has been shown that some human tumour cell lines produce large amounts of hydrogen peroxide without exogenous stimulation [33]. In addition, CYP2E1 is not the only source of H_2_O_2_ generation. Endogenous superoxide dismutase may also produce significant amounts of H_2_O_2_ at physiological pH [34]. Likewise, several kinds of oxidases, including flavine [35] or NADPH oxidase [36], are able to produce H_2_O_2_ directly in mammalian cells. These factors may contribute to the high basal level of ROS in C3A cells.

Although DDC is considered as a relatively selective inhibitor of CYP2E1, our results demonstrated that DDC suppressed the increase in intracellular ROS in LX-2 cells of both co-culture systems. In addition, DDC suppressed the increase of collagen I protein in LX-2 cells and in the culture medium of both co-culture systems. These data suggested that the suppressing effect of DDC on ROS production does not merely depend on inhibition of CYP2E1 activity. It is possible that DDC could also suppress ROS production by inhibiting SOD (superoxide dismutase) activity [37,38].

In the present study, we demonstrated that DDC significantly up-regulated the expression of MMP-1 in LX-2 cells co-cultured with C3A-2E1. Subsequently, the increased expression resulted in greater enzyme activity and a decrease in collagen I. Since the only major difference between the C3A and C3A-2E1 cells is the expression of CYP2E1, the up-regulation of MMP-1 by DDC is likely due to CYP2E1 activity. We also investigated the effect of DDC on MMP-1 in LX-2 cells co-cultured with C3A (without CYP2E1). Interestingly, although MMP-1 up-regulation was not as high as with the C3A-2E1 co-culture system, DDC could also up-regulate the expression of MMP-1 in LX-2 cells co-cultured with C3A cells (without CYP2E1). These data suggested that the effect of DDC on MMP-1 did not only depend on the inhibition of CYP2E1, and that additional mechanisms may be involved.

Since DDC inhibits ROS levels and up-regulate MMP-1 expression in both co-culture systems, it was important to explore the inhibitory effect of DDC on ROS and the stimulatory effect on MMP-1 expression. Our results show that H_2_O_2_ treatment significantly attenuated DDC-induced up-regulation of MMP-1 expression in LX-2 cells, suggesting that H_2_O_2_ inhibition by DDC partially contributed to the up-regulation of MMP-1 by DDC. In addition, catalase treatment alone, which is an antioxidative enzyme that degrades H_2_O_2_, slightly up-regulated MMP-1 expression. These findings suggest that H_2_O_2_ inhibition by DDC only plays a small role in the MMP-1 up-regulation by DDC.

MAPKs, a ubiquitous group of serine/threonine kinases, play a crucial role in transmitting transmembrane signals required for cell growth, differentiation and apoptosis. Previous studies have shown that MAPK pathways mediate MMP-1 expression induced by various stimuli [39,40]. For example, the ERK1/2 pathway mediates the activation of the MMP-1 promoter *via* an AP-1 element induced by Ras, serum, phorbol ester, insulin and oncostatin [41,42], and specific activation of ERK1/2 induces MMP-1 production [43]. However, p38 MAPK has opposing effects on *MMP-1* gene expression depending on the inducing agent. p38 MAPK activated by interleukin-1 [44], tumor necrosis factor-α [44], C2 ceramide [45] or okadaic acid [46] up-regulates MMP-1 gene expression. In contrast, activation of p38 induced by three-dimensional collagen lattices or by arsenite inhibits *MMP-1* gene expression [47,48]. These data suggest that coordinated activation of multiple MAPK pathways determines the rate of *MMP-1* gene expression. Akt, a serine/threonine protein kinase, plays a critical role in controlling the balance between apoptosis and cell survival in response to extra- and intracellular signalling [49]. Three isoforms, such as Akt1, Akt2 and Akt3, are homologous, but differ slightly in the localization of their regulatory phosphorylation sites in mammals. Akt1 is the predominant isoform in most tissues and is activated by phosphorylation on Ser^474^ and Thr^308^. Previous studies have shown that Akt activation was also involved in MMP-1 secretion [50,51].

In the present study, we found that DDC activated ERK1/2, p38 and Akt in LX-2 cells. The ERK1/2 inhibitor U0126 and Akt inhibitor T3830 suppressed the DDC-induced MMP-1 expression, suggesting that ERK1/2 and Akt are involved in this up-regulation. In addition, our findings demonstrated that DDC-mediated induction of MMP-1 was improved when treated with SB203580 (p38 MAPK inhibitor). To investigate the possible mechanisms, we observed the effect of SB203580 on the activation of ERK1/2 and Akt by DDC. Our data showed that SB203580 significantly activated ERK1/2, while inhibiting Akt activation. These results indicate that the p38 MAPK acts as a negative regulator of MMP-1 induction following DDC treatment in LX-2 cells, through ERK1/2 stimulation.

In conclusion, this is the first report demonstrating that DDC significantly induces MMP-1 expression, which results in greater enzyme activity. In LX-2 cells, this induction was partially due to the inhibition of ROS production, and was regulated in a coordinated manner by the ERK1/2, p38 and Akt pathways. When considering antifibrotic therapy, the approach should not only target the causative agent(s) of liver injury, but also focus on HSC activation status, with two complementary objectives: (a) to directly down-regulate HSC activation; (b) to increase ECM degradation [52]. Since DDC not only inhibits the ROS production that activates HSC and stimulates collagen synthesis, but also induces MMP-1 expression that degrades the collagen protein and other ECM, we believe that our findings will appeal to both researchers and clinicians.
